# Dataset for Comprehensive Analysis of Desiccation Cracks in Soils

**DOI:** 10.1038/s41597-026-06632-6

**Published:** 2026-02-27

**Authors:** Amirali Asadian, Farshid Vahedifard, Chao-Sheng Tang

**Affiliations:** 1 Department of Civil and Environmental Engineering, Tufts University, Medford, MA 02155 USA; 2 United Nations University Institute for Water, Environment and Health (UNU-INWEH), Richmond Hill, ON L4B 3P4 Canada; 3 School of Earth Sciences and Engineering, Nanjing University, Nanjing, 210023 China

**Keywords:** Natural hazards, Geology

## Abstract

Soil cracking poses significant challenges to the integrity of slopes and earthen infrastructure. This study presents a dataset for comprehensive analysis of desiccation cracks in soils (D-CRACKS), aiming to enhance the understanding of this complex phenomenon. The dataset compiles 1,000 images from laboratory tests of soil desiccation cracking collected from 41 studies in the literature. D-CRACKS is built using a Structured Query Language (SQL)-based database management system, enabling efficient data retrieval, filtering, and querying. Each image is linked to detailed metadata, including testing conditions, soil properties, boundary constraints of the tested samples, environmental conditions, and admixture properties. Following data cleaning, images were analyzed to extract key crack properties such as crack area, crack ratio, crack length, and average crack width. Statistical analyses were performed to examine variability and trends across soil types and testing conditions. D-CRACKS offers a structured and scalable foundation for investigating soil cracking and offers substantial value for future research, particularly for developing data-driven predictive models and validating physics-based numerical simulations of soil cracking.

## Background & Summary

Soil desiccation cracking causes many challenges in various fields such as civil engineering, environmental engineering, soil science, climate science, agricultural engineering, and more^1,2^. Crack formation in soil due to loss of water can alter hydraulic conductivity and porosity^3^, which significantly affects water seepage and retention. Cracking can also impact soil shear strength and pose many problems to the integrity and stability of earthen infrastructure and slopes^4,5^. In civil engineering, desiccation cracks significantly impact infrastructure resilience and stability. The formation of these cracks can compromise the foundation stability of buildings, bridges, and roads. Moreover, earthen structures such as dams, levees, and retaining walls are particularly vulnerable to issues arising from soil desiccation. As the soil loses moisture, its mechanical properties change markedly, leading to deformation and decreased strength^1,6^. Additionally, the formation of cracks alters the soil’s hydraulic conductivity and creates preferential flow paths^7^, which can exacerbate erosion and further weaken the soil’s structural stability. In agriculture, crack formation can alter seedling emergence^8^, inhibit root penetration^9,10^, and affect moisture availability^11,12^, ultimately affecting crop yield^13^. In environmental management, soil cracks can alter the permeability and hydraulic conductivity of the soil, increasing the risk of subsurface water contamination^14,15^. In archaeological sites, soil desiccation can lead to the deterioration of significant artifacts and excavation sites, affecting the long-term preservation of these sites^16,17^.

The importance of studying soil desiccation cracking has grown significantly in the context of a changing climate^18,19^. Anthropogenic climate change has been shown to exacerbate patterns of prolonged drought, heatwaves, dry-wet cycles, and freeze-thaw cycles in many regions, thereby increasing the susceptibility of soils to cracking^20^. Recent studies highlight that desiccation cracking plays a crucial role in the complex interplay of drought and greenhouse gas emissions^2^. Given these implications, it becomes imperative to elevate our understanding of desiccation cracks in the soil, not only to mitigate their adverse effects on infrastructure but also to inform strategies that address their environmental and climatic interactions.

**Fig. 1:**
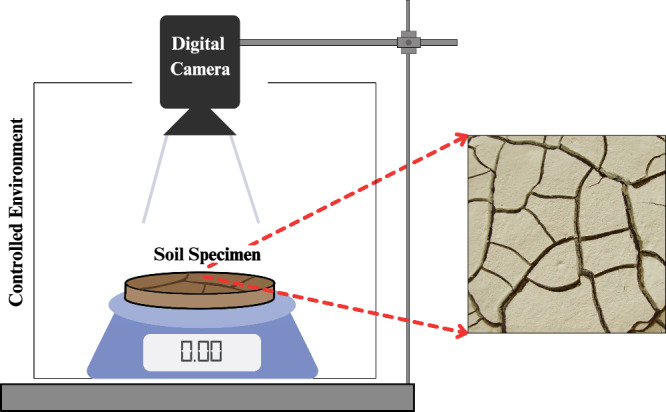
Schematic view of soil desiccation crack test.

Several studies have attempted to characterize and quantify the phenomenon of soil desiccation cracking. A large group of studies focuses on experimental approaches, using monitoring and imaging techniques such as camera imaging^21–29^, laser devices^30,31^, and X-ray computed tomography^30,32^ among other techniques. To investigate soil desiccation cracks, controlled laboratory tests are typically conducted on soil specimens, either compacted or slurry, of various geometries and materials^19,20,33^. In these tests, the specimens are subjected to drying conditions in a controlled environment, such as heat-induced or air-dry, with controlled and uncontrolled parameters such as temperature and humidity carefully monitored^34^. The specimens are placed in specially designed chambers that allow for accurate control and measurement of the drying process. Over time, the drying process is monitored, and images are captured at intervals to document the formation and progression of cracks. These images are then analyzed using image processing techniques to determine crack characteristics, including crack area, length, width, crack ratio, crack number, and fractal dimension^35^. A schematic figure illustrating a typical laboratory test setup is shown in Fig. 1.

Despite these efforts, systematic analysis of laboratory test results remains challenging, largely due to the scattered nature of the data^1,36^. The challenge of developing a unified understanding of this complex phenomenon is further complicated by the lack of integrated data collection methods across different disciplines. Few studies have explored the application of machine learning and deep learning techniques in predicting desiccation crack characteristics^37^ using quantified crack characteristics. Despite recent advancements in machine learning and data-driven models, significant progress in the field of desiccation crack analysis remains elusive. This highlights the necessity for, and the absence of, relevant and detailed datasets. Such datasets are essential for the development and application of advanced models that can accurately predict and analyze desiccation cracks.

Here, we present a dataset for comprehensive analysis of desiccation cracks in soils named D-CRACKS, providing a comprehensive dataset of laboratory testing results on soil desiccation cracks. D-CRACKS comprises 1,000 images of soil desiccation cracking from 45 studies in the literature. Built using a database management system with Structured Query Language (SQL), it enables efficient data retrieval and querying. Each image is linked to detailed metadata, including testing conditions, soil properties, boundary constraints of the tested samples, environmental conditions, and admixture properties. The images underwent data cleaning and analysis to extract various crack properties, such as crack area, crack ratio, crack length, and average crack width. Statistical analyses were conducted to examine the crack properties across the dataset. D-CRACKS provides researchers with valuable data to enhance their understanding of soil desiccation mechanisms and promote new research initiatives across multiple disciplines.

## Methods

### Data collection

In the initial step, we conducted a comprehensive literature review to identify relevant research on soil crack desiccation. Using academic search engines such as Google Scholar (scholar.google.com) and ScienceDirect (sciencedirect.com) (last accessed: November 2025), with key phrases like “soil cracking,” “soil desiccation cracking,” and “soil desiccation crack image analysis,” we identified studies containing images of desiccation cracks. The dataset was compiled following explicit inclusion criteria to ensure representativeness and reproducibility. Studies were included only if they were (i) peer-reviewed and published in reputable journals or conference proceedings; (ii) featured laboratory-based soil desiccation tests with sufficient methodological description (e.g., specimen preparation, environmental conditions, or soil properties); (iii) provided clear crack-pattern images at identifiable test stages, with resolution sufficient for analysis; (iv) offered key metadata (soil properties, environmental conditions, boundary constraints) necessary for systematic linkage. Although the dataset is not exhaustive, these criteria ensured the inclusion of high-quality, well-documented experiments representative of state-of-the-art soil cracking research. For each study included, the DOI of the article was recorded in the dataset metadata if available, and where datasets were directly available in repositories, the dataset DOI was cited in the reference table in the data to ensure full reproducibility of the data collection process.

All metadata and image-derived data included in D-CRACKS were obtained and derived from openly accessible peer-reviewed journal articles and repositories. To ensure full compliance with copyright and reuse policies, original crack photographs from published studies are not redistributed or included in the dataset. Instead, crack images were used solely as intermediate inputs to derive non-reversible representations (binary masks) for quantitative analysis. Only derived data products and reported experimental metadata are shared openly.

**Fig. 2:**
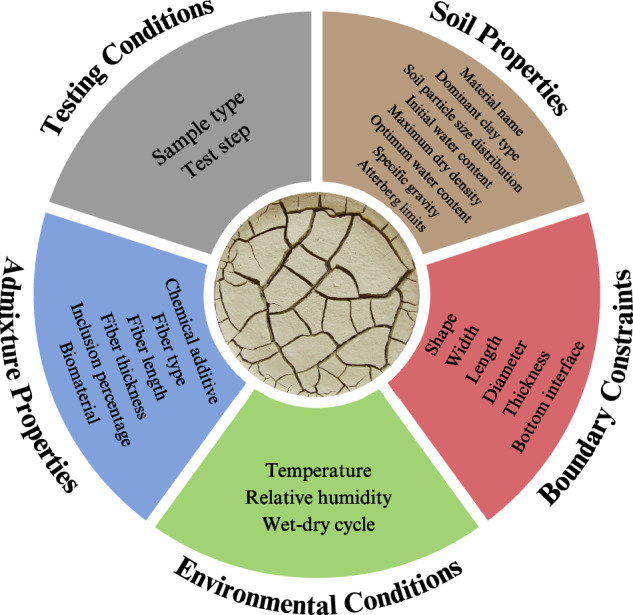
Extracted data from the literature, categorized into five groups of attributes.

The final dataset comprises 1,000 derived crack representation images from 41 independent studies across multiple research groups, offering a robust baseline for systematic analysis and machine learning applications. From these articles, we extracted pertinent data on testing conditions. We used five key groups of attributes^1^ to extract desiccation crack data: (1) testing conditions (specimen type, test step that image is taken), (2) soil properties (mineralogy, sieve analysis, initial water content, maximum dry density, Atterberg limits, etc.), (3) boundary constraints (sample shape, sample size, thickness, sample bottom interface, and vegetative cover), (4) environmental conditions (temperature, relative humidity, and wet-dry cycles), and (5) admixture properties. (Fig. 2). Simultaneously, we collected images of soil desiccation from the selected papers. We prioritized images directly available within the publications. In several cases, additional crack images were obtained directly from laboratory experiments conducted by the authors of this study; these images correspond to tests described in the associated publications but were not included in the published figures and are free of third-party copyright restrictions. Each image was cataloged alongside the conditions reported in the respective studies, such as soil type, environmental conditions, and test parameters. It is important to note that the resolution and quality of images in D-CRACKS are inherently tied to the resolution of the original publications. Thus, image quality varies and is directly correlated with the publishing standards of the source journals.

The next steps involved data cleaning and image analysis, utilizing dedicated Python libraries and specialized image processing software. We standardized image dimensions, removed obstructions, and assessed image quality using Python libraries, excluding subpar images to maintain high analytical standards. For image analysis, we employed the Crack Image Analysis System (CIAS) software^26^ (available at https://es.nju.edu.cn/climatengeo_en/main.htm), converting images to grayscale, segmenting them, and using binarization to highlight crack patterns. We refined images by removing noise and applied the Classic Skeleton algorithm and pruning processes to ensure accurate crack representation. These processed images, along with their geometric parameters, were then stored in the database.

### Data cleaning

**Fig. 3:**
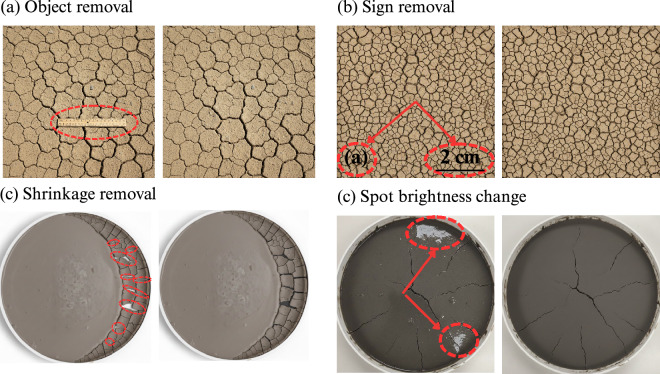
Preprocessing workflow prior to binarization, illustrated using a synthetic crack image.

The data-cleaning process began with standardizing image dimensions, including adjusting image perspectives to focus solely on the sample area of interest. This was necessary because some images were not captured perpendicular to the sample. Each image was meticulously cropped to remove extraneous spaces, ensuring consistency across the dataset. Next, a thorough visual inspection of each image was conducted to identify and remove foreign objects, such as rulers, labels, arrows, or dimension markers (Fig. 3a,b), which could interfere with the crack analysis.

In some cases, particularly during the later stages of crack formation, the cracks revealed the underlying plate material in the background with higher gray levels. This issue made the binarization process problematic, as shrinkage areas were misidentified as cracks (Fig. 3c). To address this, adjustments to brightness and saturation were applied to enhance crack visibility and ensure accurate analysis (Fig. 3d). To objectively evaluate image quality, we utilized Python’s imaging libraries, including OpenCV and the Pillow library. These tools allowed us to retrieve each image’s width, height, resolution, and file size, ensuring that the crack analysis results could be accurately converted to real-size measurements. Finally, the data extracted from various studies were harmonized into a consistent format with uniform terminology and measurement units. Measurements within each column were standardized to consistent units, details of which are provided in the README file.

### Image analysis

Following the data-cleaning phase, we utilized the CIAS software^26^, a specialized tool operating within the MATLAB environment, for the automatic identification and quantitative analysis of soil desiccation cracks. This software is designed to process high-resolution images and extract detailed geometric information pertinent to the analysis of crack patterns. The CIAS software provides different outputs such as total crack length, average crack width, crack area, crack ratio, crack segments, and fractal dimensions.

Initially, upon loading the images into the CIAS software, we converted them into grayscale. Since cracks are typically darker and have lower gray levels, this step allows for differentiation based on varying gray levels^38^. This conversion is essential for the subsequent segmentation process. Following grayscale conversion, we proceeded with binarization, a step aimed at highlighting the crack patterns within the images while minimizing the inclusion of non-crack features. To ensure optimal crack delineation for every sample, we employed an adaptive approach that tested two distinct binarization methodologies. The first methodology involved traditional thresholding techniques available within the CIAS environment, allowing us to select a specific gray-level threshold for optimal differentiation. The second methodology leveraged the Gemini API, a large multimodal model, for automated, context-aware segmentation. For the Gemini approach, we used a specific prompt requesting the model to convert the image into a strictly binary output (black cracks on a white background) while filtering out noise.

After processing each image using both the traditional and the AI-based methods, the resulting binary image with the clearest distinction between crack and clod areas was manually selected for use in subsequent analysis, thus mitigating the subjectivity of relying on a single binarization technique. The resulting binary image served as the basis for identifying noise within the areas of cracks and clods. After binarization, image noise can create white spots within the cracks and black spots within the clods. To refine the processed image, we employed a seed-filling algorithm to remove these noises, allowing for more accurate delineation of clods and cracks^29^. The extraction of the crack network’s medial axis was performed using the Classic Skeleton algorithm^21^. This algorithm reduces the binary image of the crack network to a one-pixel-width representation, preserving the geometrical properties of the cracks^29^. Given that skeletonization can introduce parasitic branches, which are extraneous elements that do not represent actual cracks, we applied a pruning process to clean up the skeletonized image^39^. The pruning threshold was set based on the maximum crack width, as it is generally larger than the length of any parasitic branches, ensuring the retention of only the significant crack structures for further analysis. By identifying clods, cracks, and nodes, geometric parameters such as crack segment numbers, crack ratio, crack area, total crack length, average crack width, and fractal dimension can be provided by CIAS.

**Fig. 4:**
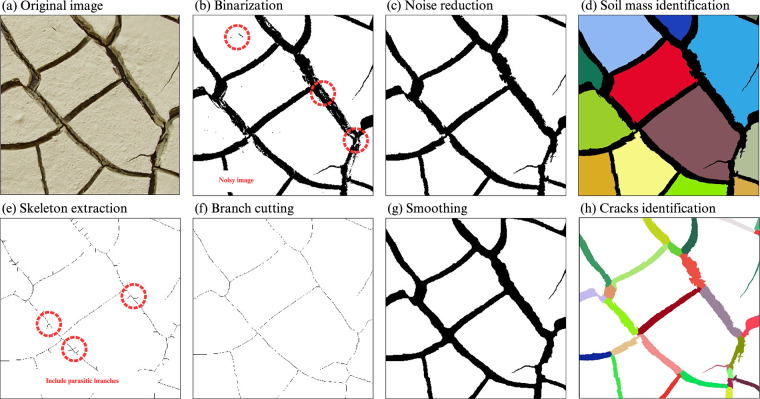
Steps of the crack identification process in the CIAS software.

The overall data-cleaning and processing workflow is illustrated in Figs. 3, 4. Figure 3 demonstrates key preprocessing steps with representative examples (object removal, brightness adjustment, and shrinkage correction), while Fig. 4 outlines the sequential image-processing steps applied using CIAS software. Together, these figures provide a visual flow chart of the complete data-preparation pipeline.

### Database preparation

To effectively manage and store the processed image data, we employed the PostgreSQL database management system, interfaced through pgAdmin. This setup allowed for robust data manipulation and querying capabilities essential for handling large datasets. Initially, SQL scripts were written to construct the necessary tables within D-CRACKS. An important step in this process was selecting appropriate type modifiers for each column. This selection was guided by the nature of the data (e.g., numerical, textual, binary) to ensure that data storage is optimized.

Subsequently, constraints were defined and applied to each table. These constraints are necessary for maintaining the database’s integrity and accuracy. This systematic application of constraints preserves the data integrity and enhances the robustness of D-CRACKS, making it well-suited for future expansions and additional data integrations. In the context of our research on soil crack desiccation, employing a database architecture offers significant advantages. First and foremost, it ensures consistency across multiple datasets. This is essential for maintaining the accuracy of our analyses as the database scales with additional data from ongoing and future experiments. Additionally, the scalability and security features of databases ensure that our data remains secure as the scope of our research expands, which is vital for long-term studies and collaborations with other researchers.

In total, eight internal tables were generated based on related attributes, including testing conditions, soil properties, boundary constraints, environmental conditions, admixture properties, references, crack properties (CIAS results), and image properties. Table 1 summarizes the key data records associated with D-CRACKS. Each record corresponds to a specific article, detailing the source and the number of images extracted from each article. Each data record, sourced from peer-reviewed articles, is documented and cited in the dataset.

**Table 1:** List of articles and number of images extracted from each article.

Study	N	Study	N	Study	N
Liu *et al*., 2024^51^	387	Wang *et al*., 2025^62^	15	Zhuo *et al*., 2024^74^	6
Zeng *et al*., 2022^19^	97	Yang *et al*., 2025^63^	15	Li *et al*., 2024^75^	6
Liu *et al*., 2024^52^	72	Zeng *et al*., 2019^18^	14	Tang *et al*., 2001^27^	5
Vail *et al*., 2020^53^	36	Wang *et al*., 2025^64^	12	Tang *et al*., 2011^27^	4
Wei *et al*., 2024^54^	27	Tang *et al*., 2011^65^	12	Tang *et al*., 2013^76^	3
Tang *et al*., 2024^20^	23	Tang *et al*., 2007^66^	12	Zeng *et al*., 2019^77^	3
Guo *et al*., 2025^55^	20	Tang *et al*., 2018^67^	11	Lakashmikhanta *et al*., 2018^78^	3
Gebrenegus *et al*., 2011^30^	20	Panayiotopoulos *et al*., 2009^68^	10	Tang *et al*., 2016^73^	2
Zhao *et al*., 2025^56^	20	Tay *et al*., 2001^69^	10	Cui *et al*., 2014^79^	2
Liu *et al*., 2020^57^	20	Hirobe *et al*., 2016^70^	8	Vogel *et al*., 2005^38^	2
Wang *et al*., 2025^58^	19	Luo *et al*., 2023^71^	8	Tang *et al*., 2018^67^	1
Tang *et al*., 2012^59^	18	Gao *et al*., 2025^72^	8	Rayhani *et al*., 2008^45^	1
Tollenaar *et al*., 2017^60^	17	Yesiller *et al*., 2000^41^	7	Tang *et al*., 2010^80^	1
Wan *et al*., 2019^61^	16	Tang *et al*., 2016^73^	7	Tests by the Authors (not published previously)	20

## Data Records

All data files related to D-CRACKS are available in the Figshare repository^40^ (10.6084/m9.figshare.28441910). The repository contains the following files and folders:

- D-CRACKS.csv: The primary dataset containing all extracted and processed information. This file includes 51 columns representing attributes such as testing conditions, soil properties, boundary constraints, environmental conditions, admixture properties, crack properties, and image properties.
- README.txt: A detailed description of the dataset structure, including definitions of all column headings, units, and variable names.
- /images_binary/: A folder containing binary crack representations generated by CIAS software for crack identification and quantification.

Each data record contains multiple columns, representing specific variables measured during the study. The descriptions of these columns are provided in Table 2. The dataset consists of 1 general table (8 internal tables) and 51 columns. The following subsections provide a detailed description of the key attributes of six tables (i.e., testing conditions, soil properties, boundary constraints, environmental conditions, admixture properties, crack properties). The other two tables include the citation to the original reference as well as the properties of acquired and analyzed images.

**Table 2:** Summary of columns of the CSV file consists of the column name, description, and its unit.

Category	Header	Description	Units
ID	id	Unique ID of the image	—
Test properties	sample_type	Compacted, slurry or in-situ	
	test_step	The test step in which the image is taken	—
Soil properties	material_name	Material commercial name	
	uscs	Unified soil classification system name of sample	—
	clay_type	Dominant clay type	—
	coarse_percentage	Coarsed-grain soil (sand and gravel) fraction	%
	silt_percentage	Silt fraction	%
	clay_percentage	Clay-size fraction	%
	fine_content	Fine-grained soil (silt and clay) fraction	%
	initial_water_content	Initial water content	%
	water_content	Water content in which the image is taken	%
	maximum_dry_density	Maximum dry density	g/cm^3^
	optimum_water_content	Optimum water content	%
	specific_gravity	Specific gravity (Gs)	—
	liquid_limit	Liquid limit (LL)	%
	plastic_limit	Plastic limit (PL)	%
	plastic_index	Plastic index (PI)	%
	shrinkage_limit	Shrinkage limit (SL)	%
Boundary constraints	shape	Shape of the sample (circle, square, or rectangle)	—
	length	Length of the sample	mm
	width	width of the sample	mm
	diameter	Diameter of the sample	mm
	thickness	Thickness of the sample	mm
	bottom_interface	Bottom material of the sample	—
	vegetative_cover	Vegetative cover of the soil sample	—
Environmental conditions	temperature	Test temperature	Celsius (degree)
	relative_humidity	Relative humidity of test condition	%
	wet_dry_cycle	The Wet-dry cycle in which the image is taken	—
Admixture properties	additive_material	Additive material	—
	fiber_type	Fiber type	—
	fiber_length	Fiber length	mm
	fiber_thickness	Fiber thickness	mm
	inclusion_percentage	Fiber inclusion percentage	%
	biomaterial	biomaterial	—
Publication details	doi	Digital Object Identifier	—
	citation	Study citation	—
	paper_title	Study title	—
	year_published	The year the study published	—
crack properties	crack_area	Total crack area from image analysis	mm^2^
	crack_ratio	Crack ratio from image analysis	—
	total_crack_length	Total crack length from image analysis	mm
	average_crack_width	Average crack width from image analysis	mm
	crack_segments	Number of crack segments from image analysis	—
	fractal_dimension	Fractal dimension from image analysis	—
Image properties	img_width	Width of image	Pixel
	img_height	Height of image	Pixel
	dpi	Image resolution	Dots per inch (DPI)
	snr	Signal-to-noise ratio of image	—
	laplacian_variance	Laplacian variance of image	—
	file_size_kb	Image size	KB
	note	Note	—

## Technical Validation

### Data completeness and coverage

#### Testing conditions

The samples in soil desiccation crack testing can be prepared in two distinct ways: slurry and compacted samples. Slurry samples, the more common method, involve mixing the soil with water to form a thick, homogeneous mixture, which is then allowed to settle and dry under controlled or natural conditions. This method is particularly useful for observing crack formation from an initially saturated state. On the other hand, compacted samples are created by compressing soil at a specified water content to achieve a desired density, simulating *in-situ* soil conditions. These samples help in understanding the cracking behavior of soils as they lose moisture and shrink. Out of a total of 1,000 samples, 458 (45.8%) are compacted, and 486 (48.6%) are prepared as slurry. The images in the dataset were taken during various stages of the desiccation process, capturing the progression of crack formation from the initial drying phase to the point where the soil reaches a stable cracked state. In total, 411 images are from the final crack formation step.

#### Soil properties

Soil properties in D-CRACKS indicate that 89.90% of the data includes clay percentage, making it a valuable attribute for investigating the relationship between clay content and crack properties. Similarly, 92.20% of the data includes a Liquid Limit (LL) value, and 89.70% includes a Plastic Limit (PL) value.

**Fig. 5:**
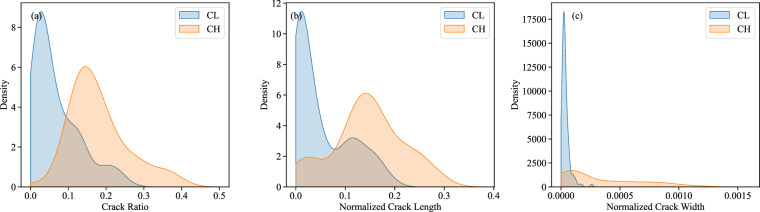
Effect of low and high-plasticity clay on crack properties. (**a**) distribution of crack ratio, (**b**) distribution of normalized crack length, and (**c**) distribution of normalized crack width.

The soil properties in D-CRACKS are classified according to the Unified Soil Classification System (USCS), specifically focusing on CH (high plasticity clay) and CL (low plasticity clay). CH soils are characterized by high clay content and significant plasticity, leading to considerable shrinkage and crack formation upon drying. In contrast, CL soils exhibit lower plasticity and moderate shrinkage, resulting in less extensive cracking ^41^. In our dataset, 880 samples are USCS classified, and out of these 880, 670 are CL and 210 are CH. The main observed patterns are summarized in Fig. 5, which illustrates differences in crack ratio, crack length, and crack width between CL and CH soils. The CH soil samples’ crack ratio is clustered around 0.1 to 0.3, while the CL soil samples are clustered around 0.05 to 0.15. This clustering also holds for normalized crack length and crack width, which were obtained by dividing each measurement by the corresponding sample area. Having 88 percent of images classified as CL and CH will be a great feature for machine learning applications. Because crack parameters such as area, total length, and crack width are inherently dependent on sample size, we normalized these values by the specimen area. Crack ratio, normalized length, and width enable direct comparison across samples of different sizes and ensure that observed relationships are not artifacts of scale effects in these comparisons.

**Fig. 6:**
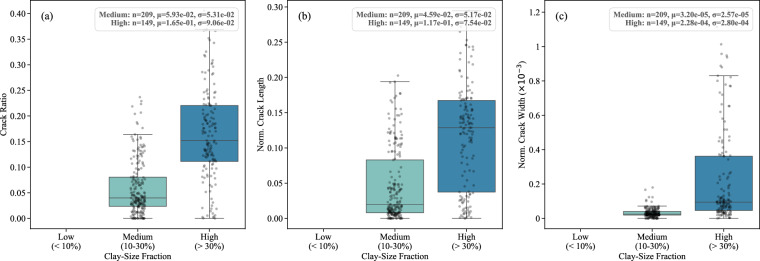
Distribution of crack characteristics across clay-size fraction categories: (**a**) crack ratio, (**b**) normalized length, and (**c**) normalized width. Subplots show data for Low, Medium, and High ranges, including descriptive statistics.

The clay-size fraction significantly impacts crack formation. A high clay-size fraction typically increases the soil’s shrinkage potential, promoting extensive crack networks^42^, whereas higher silt content can reduce cracking due to the lesser shrinkage potential of silty soils., Clay-size fraction, categorized here into Low (<10%), Medium (10-30%), and High (>30%) groups, also influences the desiccation process, as higher clay fraction generally enhances the soil’s ability to retain water, affecting the timing and pattern of crack development^43^. The plot shown in Fig. 6 corresponds to the final stable crack formation stage, when specimens had reached equilibrium after drying, thereby reducing variability from transient water content differences. Because the dataset integrates studies with varying boundary conditions (e.g., sample geometry, thickness, and bottom interfaces), crack parameters were normalized by sample area and analyzed in aggregate to identify broad relationships between clay-size fraction and crack properties. To provide a quantitative summary, each subplot includes descriptive statistics: sample size, mean, and standard deviation. We note that these boundary condition differences contribute to scatter in the data and may mask subtler trends, and therefore, readers should interpret these relationships with this limitation in mind. The Liquid Limit (LL), Plastic Limit (PL), and Plasticity Index (PI) are important soil properties that significantly influence desiccation crack characteristics. These properties determine the soil’s consistency and behavior under varying moisture conditions, thereby affecting the formation and progression of cracks during the drying process^3,44^.

The Liquid Limit (LL) is the moisture content at which soil transitions from a plastic to a liquid state. Higher LL values indicate soils that can retain more water before transitioning to a liquid state, typically associated with high plasticity clays. Soils with higher LL values tend to have higher crack ratios, as they shrink more upon drying due to their higher water retention capacity^45^. The Plastic Limit (PL) is the moisture content at which soil transitions from a semi-solid to a plastic state. It provides insight into the soil’s workability and its behavior at lower moisture contents. Soils with lower PL values are more prone to cracking, as they transition to a semi-solid state at lower moisture contents, leading to earlier and more pronounced crack formation. These soils typically exhibit narrower and shorter cracks, as they do not undergo extensive volumetric changes compared to soils with higher PL values. The lower PL values trigger earlier desiccation, resulting in the development of cracks at lower moisture levels^1,45^.

**Fig. 7:**
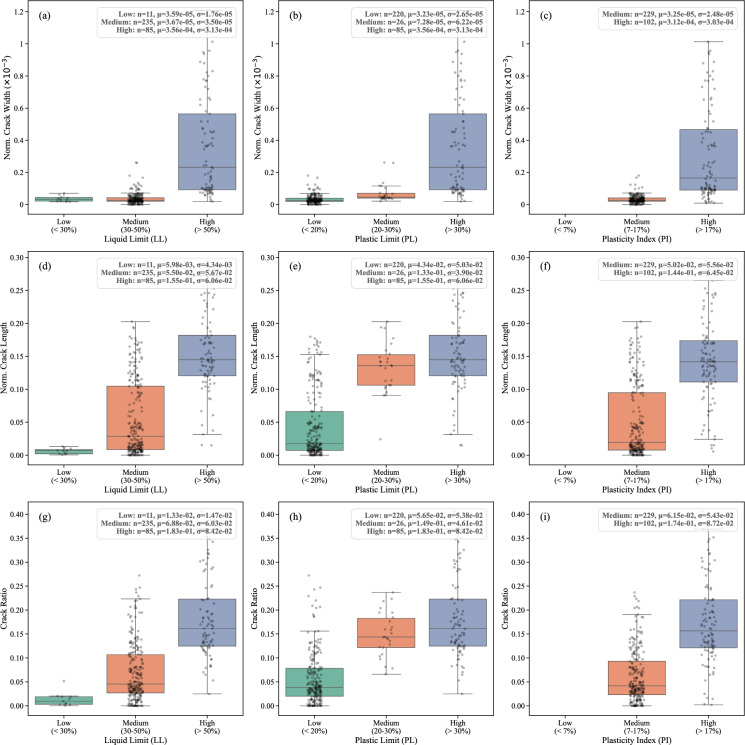
Distribution of crack characteristics across Atterberg limit categories. Results are grouped into Low, Medium, and High ranges for Liquid Limit (LL), Plastic Limit (PL), and Plasticity Index (PI). Subplots illustrate (**a–c**) normalized crack width, (**d–f**) normalized crack length, and (**g–i**) crack ratio.

As expected, our analysis of D-CRACKS demonstrates that soils with higher LL and PI values tend to develop more extensive and complex crack networks. Fig. 7 groups these results into Low, Medium, and High categories for each limit (LL: <30%, 30-50%, >50%; PL: <20%, 20-30%, >30%; PI: <7%, 7-17%, >17%), illustrating the distribution of (a–c) normalized crack width, (d–f) normalized crack length, and (g–i) crack ratio. High LL and PI values are particularly indicative of soils that undergo significant volumetric changes during drying, leading to higher crack ratios and larger crack dimensions. Understanding these relationships is necessary for predicting and managing soil desiccation cracking, especially in high-plasticity soils that are prone to extensive cracking under drying conditions. A summary of soil properties statistics in D-CRACKS is presented in Table 3. While it is well established that higher plasticity soils are more prone to cracking, the scale of D-CRACKS allows us to provide reproducible, quantitative ranges that can be directly applied in predictive modeling. For example, crack ratios for CH soils consistently clustered between 0.10–0.30, while those for CL soils clustered between 0.05–0.15 across multiple independent studies. These quantifiable thresholds extend beyond qualitative descriptions and can serve as benchmarks for future research.

**Table 3:** Summary of descriptive statistics of soil properties in the dataset.

Unit	Sand	Silt	Clay	Initial Water Content	Maximum Dry Density	Optimum Water Content	LL	PL	PI	SL
	(%)	(%)	(%)	(%)	(g/cm^3^)	(%)	(%)	(%)	(%)	(%)
Mean	5.37	63.06	33.67	107.33	1.70	16.54	55.26	23.83	32.19	11.11
Std.	10.85	21.92	18.85	62.14	0.04	1.40	49.06	8.54	44.81	0.82
Min	0.65	11.00	22.00	15.00	1.66	11.00	23.20	10.70	11.66	9.70
Max	83.30	76.00	86.70	170.00	2.01	32.00	276.00	42.00	239.00	14.00
Availability Percentage	71.90	83.80	89.80	34.30	63.30	63.60	92.20	89.70	87.50	20.40

#### Boundary Constraints

**Fig. 8:**
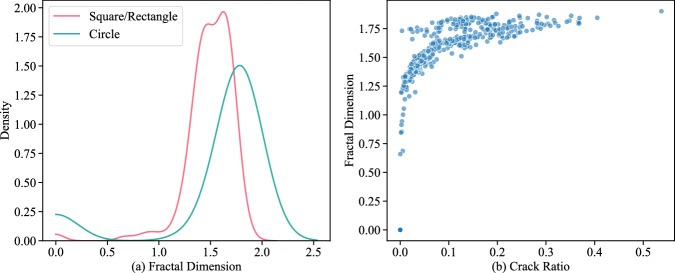
(**a**) Relationship of soil sample shape and fractal dimension, and (**b**) Increasing trend of fractal dimension with increase in crack ratio.

The geometry of the sample dictates the stress distribution as the soil dries, thereby influencing the initiation and propagation of cracks. The distinct shapes impose different stress fields^46^. The complexity of crack networks can be quantified using fractal dimension analysis, which measures how completely the cracks fill the space within the sample. Different shapes may result in varying fractal dimensions, reflecting differences in crack complexity. In our dataset, circular samples exhibit slightly larger fractal dimensions, though the differences are not substantial. Additionally, as the crack ratio increases, the fractal dimension also increases (Fig. 8).

The soil samples’ width, length, and diameter are interconnected parameters that substantially impact the cracking behavior^46^. Wider and longer samples provide a larger surface area exposed to drying, which increases the potential for crack formation. Several studies have explored the influence of sample size on crack properties^47^. The thickness of the soil sample affects both the depth and width of the cracks. Thicker samples tend to retain moisture for a longer period, which delays crack formation^36^. The vertical stress distribution within the soil is influenced by the sample’s thickness, affecting the development of deep cracks that penetrate through the entire sample. The relationship between thickness and cracking helps to understand how moisture gradients and vertical stresses interact during the drying process^26^.

The bottom interface, where the soil sample rests, also plays an important role in crack formation. The material and texture of this interface can affect how the soil shrinks and cracks. A rough or porous bottom interface increases adhesion between the soil and the base, influencing the shrinkage behavior and crack pattern. Conversely, a smoother bottom interface allows for more uniform shrinkage, resulting in more evenly distributed cracks^48^. The interaction between the soil and the bottom interface is essential for determining the overall crack pattern and extent, highlighting the importance of considering base conditions in desiccation studies. In our dataset, the available bottom interfaces are wood, metal with a perforated base, glass, sandpaper, copper tray, perforated plastic, acrylic, steel, plastic, and existing soil.

#### Environmental conditions

**Fig. 9:**
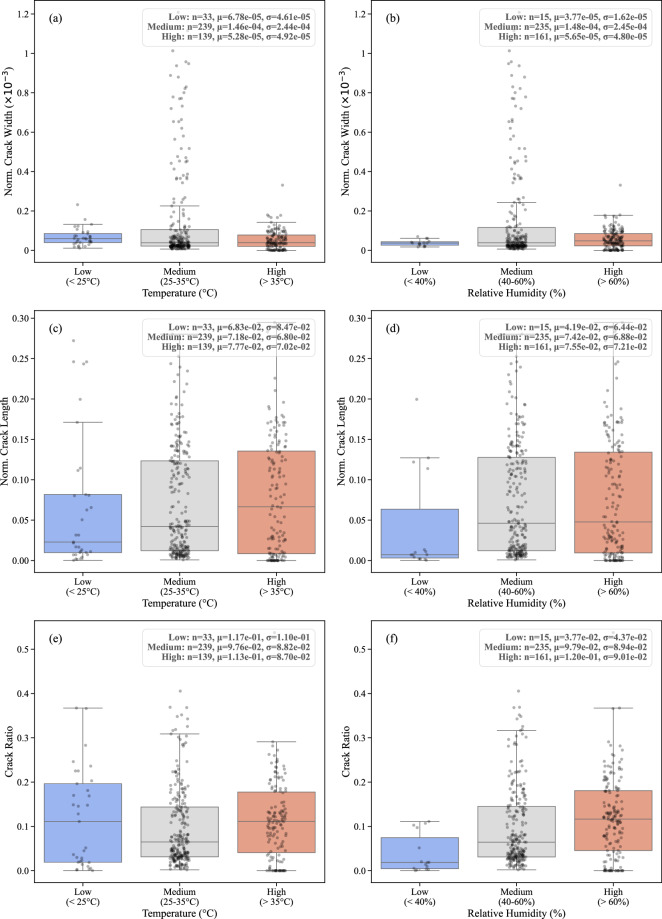
Distribution of crack characteristics across environmental factor categories. Subplots illustrate (**a,b**) normalized crack width, (**c,d**) normalized crack length, and (**e,f**) crack ratio across defined ranges of temperature.

**Fig. 10:**
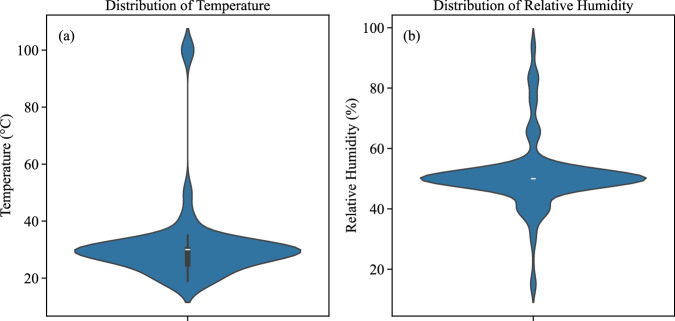
Violin plot of temperature and relative humidity in the dataset.

Temperature is a crucial environmental factor affecting soil desiccation. Higher temperatures accelerate the evaporation of water from the soil, leading to faster drying rates. This rapid moisture loss induces greater shrinkage and stress within the soil matrix, resulting in more extensive cracking. Soils exposed to elevated temperatures tend to exhibit higher crack density and larger total crack areas. Additionally, higher temperatures can cause wider and longer cracks due to the increased volumetric changes that occur during drying. By analyzing the temperature data in our dataset, categorized into Low (<25°C), Medium (25-30°C), and High (>30°C) ranges, we can quantify the relationship between temperature and crack characteristics, revealing that increased temperatures correlate with more severe cracking. Relative humidity (RH) influences the rate at which soil loses moisture to the atmosphere. Lower RH levels advance faster evaporation, leading to quicker desiccation and crack formation. In contrast, higher RH levels slow down the drying process, resulting in less extensive cracking. As shown in Fig. 9, these environmental factors are grouped into categories (RH Low: <40%, Medium: 40-60%, and High: >60%) to illustrate the distribution of (a–b) normalized crack width, (c–d) normalized crack length, and (e–f) crack ratio. Along with Fig. 9 which shows the correlation between crack characteristics with changes in temperature and relative humidity in our dataset, Fig. 10 shows the availability of data for temperature and relative humidity throughout the dataset.

Wet-dry cycles, where soil alternates between wetting (rehydration) and drying phases, are crucial for understanding soil behavior under natural climatic conditions. D-CRACKS has 331 samples subjected to wet-dry or freeze-thaw tests. These cycles significantly impact soil desiccation crack characteristics. Initially, crack patterns tend to be linear with numerous small branches^15^. However, after three wet-dry cycles, the extent of cracking stabilizes and does not change significantly^49^. Soils with higher clay content and plasticity index exhibit greater volumetric shrinkage and more extensive cracking after repeated cycles^11^. Irreversible changes in soil fabric occur during the first drying cycle, affecting subsequent cracking behavior^50^. Ultimately, soils can reach an equilibrium state where further wet-dry cycles have minimal impact on crack development^27^.

#### Admixture properties

Admixture properties play a significant role in influencing the desiccation cracking behavior of soils. Admixtures, including materials like fibers, polymers, and chemical stabilizers, are incorporated into the soil to enhance its structural integrity and reduce its susceptibility to cracking. Fibers, for example, can reinforce the soil matrix, distributing stresses more evenly and preventing the formation of large, continuous cracks. Polymers and chemical stabilizers can alter the soil’s water retention properties and reduce shrinkage, thereby minimizing crack development. The presence and type of admixture, along with its concentration, significantly impact the extent, width, and pattern of cracks that form during the drying process. Understanding the effects of various admixtures is crucial for developing effective soil stabilization techniques and for predicting the behavior of soil in various environmental and engineering applications. The dataset records the type and amount of admixture used in each sample, consisting of 578 tests with admixture properties that provide insights into how these additives influence soil cracking under different conditions.

### Statistical distribution of crack parameters

**Fig. 11:**
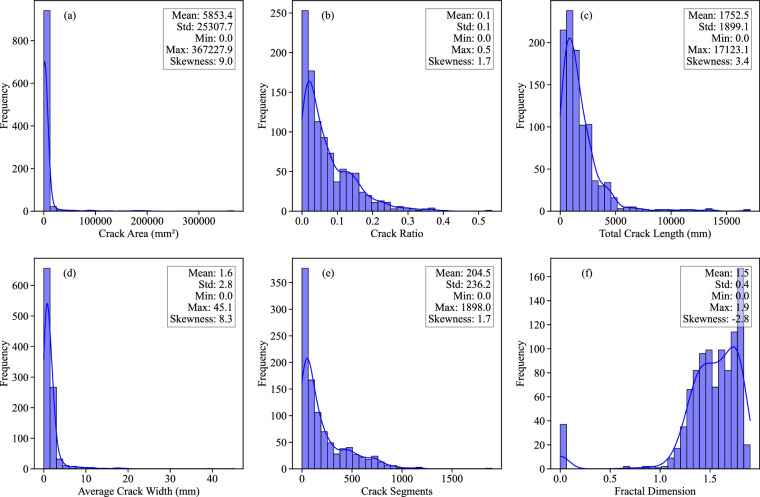
Histograms of (**a**) crack area, (**b**) crack ratio, (**c**) total crack length, (**d**) average crack width, (**e**) number of crack segments, and (**f**) fractal dimension from image analysis results.

The distributions of crack properties in D-CRACKS exhibit significant variability across different metrics, as shown in Fig. 11. The histograms reveal that total crack length (Fig. 11c) follows a highly right-skewed distribution (Skewness: 3.4), with most values clustered well below the mean of  1752.5% mm, and a maximum value extending up to 17,123.1 mm. This indicates that while most cracks are relatively short, some samples contain exceptionally long and interconnected crack networks.

Similarly, crack area (Fig. 11a) displays strong right skewness (skewness: 9.0), with a large mean of 5853.4 mm^2^ and a high maximum value reaching 367227.9 mm^2^. This suggests that the majority of samples have small cracked areas, but the presence of outliers with significantly large cracks drives the mean upward.

The average crack width (Fig. 11d) also follows a strongly right-skewed pattern (skewness: 8.3). While most values are clustered below 5 mm (mean: 1.6 mm), the distribution extends up to a maximum of 45.1 mm. This suggests that narrow cracks are common, but certain samples exhibit substantially wider cracks.

The crack ratio (Fig. 11b), representing the proportion of the cracked area to the total sample area, has a more moderate right skew (skewness: 1.7). The number of crack segments (Fig. 11e) follows a positively right-skewed distribution (skewness: 1.7), with most samples containing relatively few segments. However, the distribution extends to a maximum of 1898 segments, highlighting the existence of samples with highly complex and fragmented cracking patterns.

Lastly, fractal dimension (Fig. 11f), a measure of crack pattern complexity, exhibits a notable left-skewed pattern (skewness: −2.8. The distribution shows a clustering of values predominantly between 1.0 and 2.0, peaking toward the high end (near the maximum of 2.00 and the mean of 1.5. This clustering of higher complexity values indicates that most samples have crack patterns that utilize the available space efficiently. A detailed summary of crack property statistics is presented in Table 4.

**Table 4:** Summary of crack characteristics statistics.

	Crack Area	Crack Ratio	Total crack length	Average crack width	Crack segments	Fractal Dimension
Unit	mm^2^		mm	mm	—	—
Mean	5853.4	0.07	1752.5	1.6	204.5	1.5
Std.	25307.7	0.07	1899.1	2.8	236.2	0.4
Min	0	0	0	0	0	0
Max	367227.9	0.54	17123.1	45.1	1898.0	1.9

### Validation of image processing methodology

 In this study, several validation processes were implemented to create D-CRACKS. All the selected studies underwent peer review in reputable journals or conference proceedings, guaranteeing initial scientific validation. Throughout the data collection step, only data that had sufficient methodological clarity, adequate measurement availability, and numerical consistency (e.g., measured in standard units) were included. Non-sample visual elements (e.g., rulers, arrows, dimension markers, or labels) were excluded during preprocessing using standardized procedures applied to generate derived image representations. To validate that crucial crack information was not unintentionally removed or added, derived crack representations were independently reviewed by another author and cross-checked against reported descriptions and measurements in the source studies.

A subset of the images in our dataset included published crack measurements (e.g., crack ratio, average crack width, or total crack length) reported by the original authors. We leveraged these data to cross-validate the output of the CIAS software. For each image, the crack characteristics extracted by CIAS were directly compared to the corresponding values from the original publication. The comparison demonstrated close agreement. Any discrepancies could be attributed to subtle variations in image resolution, binarization thresholds, or differences in crack identification and segmentation. Any identified discrepancies will be documented and addressed in subsequent releases of the dataset.

Because the quality of images varied across the source publications, there is some variability in the precision of derived crack parameters. To minimize these effects, preprocessing procedures were standardized prior to binarization, and image-quality metrics (e.g., resolution, file size, signal-to-noise ratio, Laplacian variance) as metadata in the CSV file, allowing users to assess or filter by image quality. Nevertheless, very fine cracks near the resolution limit may be underestimated, and the fractal dimension is particularly sensitive to image sharpness. Users are encouraged to consider the image-quality metadata when conducting analyses that require high precision.

A systematic check for missing or out-of-range values was performed. Where the original publication did not report a property, a NULL entry was placed to avoid imputation. Extreme outliers (e.g., large crack widths) were verified against their source to confirm whether they were typographical errors or genuine results. Relationships such as “increasing clay content leads to higher crack ratio” and “higher plasticity index yields more extensive cracking” were verified using standard soil mechanics principles and previously documented trends. All shared data products consist of derived crack representations, quantitative crack metrics, and associated metadata, ensuring reproducibility without redistribution of original crack photographs. This comprehensive release ensures that other researchers can reproduce or independently assess the results.

## Usage Notes

D-CRACKS is provided as a CSV file containing 51 attributes and accompanying derived crack representations (binary formats). Researchers can easily import the CSV file into Python, R, or MATLAB for statistical analysis or machine learning tasks. To enable machine learning workflows, the CSV file can be combined with the corresponding images by adding a column containing the file paths for the images, allowing straightforward integration into data frames for analysis. For example, in Python, libraries such as pandas can be used to create a data frame containing image file paths alongside attributes and crack characteristics to create predictive models or feature extraction.

The primary contribution of this work is the creation of a large-scale, standardized, and openly accessible dataset. Because D-CRACKS is systematically structured and accompanied by rich metadata, it provides a robust foundation for future studies that aim to integrate physics-based understanding with data-driven methods. In particular, the dataset enables the application of AI/ML techniques to uncover complex, non-linear relationships governing soil cracking, enhance predictive capabilities, and support the development of next-generation analytical tools. Moreover, the detailed crack characteristics and experimental metadata make D-CRACKS well-suited for validating physics-based numerical simulations of soil cracking, offering a unique benchmark for evaluating model performance under diverse soil types and environmental conditions.

D-CRACKS currently emphasizes mostly tests of CH and CL soils, reflecting dominant trends in the literature; this may limit direct generalization to other soil types such as silts, sands, or compacted soils with mixed mineralogy. This concentration, however, aligns with the fact that CH and CL soils are both the most commonly investigated and the most susceptible to desiccation cracking. We note that the dataset was compiled through an extensive review of the literature, and only studies meeting explicit inclusion criteria for methodological detail, image quality, and metadata availability were selected, offering the most comprehensive dataset of its kind. As an openly available and expandable resource, D-CRACKS is designed to grow through future community contributions, progressively improving its diversity and representativeness.

## Data Availability

All data files related to D-CRACKS are available in the Figshare^40^ repository under 10.6084/m9.figshare.28441910.

## Code availability

For image analysis, we employed the Crack Image Analysis System (CIAS), a third-party MATLAB-based tool that is freely available at https://es.nju.edu.cn/climatengeo_en/main.htm. Users may download and install CIAS directly from the developer’s website.
